# Roles of receptor‐interacting protein kinase 1 in SH‐SY5Y cells with beta amyloid‐induced neurotoxicity

**DOI:** 10.1111/jcmm.17095

**Published:** 2022-02-02

**Authors:** Hong‐Hao Chan, Chee‐Onn Leong, Chooi‐Ling Lim, Rhun‐Yian Koh

**Affiliations:** ^1^ School of Postgraduate Studies and Research International Medical University Kuala Lumpur Malaysia; ^2^ Department of Life Sciences School of Pharmacy International Medical University Kuala Lumpur Malaysia; ^3^ Division of Applied Biomedical Sciences and Biotechnology School of Health Sciences International Medical University Kuala Lumpur Malaysia

**Keywords:** Alzheimer's disease, necroptosis, RIPK1, SH‐SY5Y

## Abstract

Alzheimer's disease (AD), the major cause of dementia, affects the elderly population worldwide. Previous studies have shown that depletion of receptor‐interacting protein kinase 1 (RIPK1) expression reverted the AD phenotype in murine AD models. Necroptosis, executed by mixed lineage kinase domain‐like (MLKL) protein and activated by RIPK1 and RIPK3, has been shown to be involved in AD. However, the role of RIPK1 in beta‐amyloid (Aβ)‐induced necroptosis is not yet fully understood. In this study, we explored the role of RIPK1 in the SH‐SY5Y human neuroblastoma cells treated with Aβ 1–40 or Aβ 1–42. We showed that Aβ‐induced neuronal cell death was independent of apoptosis and autophagy pathways. Further analyses depicted that activation of RIPK1/MLKL‐dependant necroptosis pathway was observed *in vitro*. We demonstrated that inhibition of RIPK1 expression rescued the cells from Aβ‐induced neuronal cell death and ectopic expression of RIPK1 was found to enhance the stability of the endogenous APP. In summary, our findings demonstrated that Aβ can potentially drive necroptosis in an RIPK1‐MLKL‐dependent manner, proposing that RIPK1 plays an important role in the pathogenesis of AD.

## 1 INTRODUCTION

Alzheimer's disease (AD) is a progressive neurodegenerative disease, which causes dementia. Patients usually suffer from memory loss, which disrupts their quality of life. Post‐mortem of AD’s brains generally depicted abnormal deposition of beta‐amyloid (Aβ) plaques and tau protein tangles, and these abnormal protein aggregations are believed to be main culprit of the AD pathogenesis.

Aβ is an end product of amyloid precursor protein (APP) through amyloidogenic processing cleaved by β‐ and γ‐secretase.^1^, ^2^ Emerging documentations show that the secretion of Aβ‐degrading enzymes such as insulin‐degrading enzyme and neprilysin are decreased with ageing, and this could disturb the homeostasis of Aβ and causes Aβ deposition and amyloid plaque formation, ultimately leading to neuronal cell death.^3^, ^4^ Symptomatic treatments which include cholinesterase inhibitors and N‐methyl‐D‐aspartate receptor non‐competitive antagonists are currently available to diminish AD symptoms. Therefore, discovering an effective therapeutic approach to block or modify the pathogenesis of AD is much needed to enhance the life quality of AD patients and caregivers.

Previous effort showed the potential role of receptor‐interacting protein kinases (RIPK) in amyloid signalling, in which amyloidogenic fibrils were found in cells expressing the RIPK1/RIPK3 complex required for programmed necrosis.^5^ RIPK1 has been implicated in cell survival, apoptosis and necroptosis modulation, and is highly expressed by microglial cells in human AD brains.^6^ Inhibition of RIPK1 expression reverted AD phenotypes in the APP/PS1 transgenic mouse model and enhanced Aβ degradation by murine microglial cells *in vitro*.^7^ Additionally, Necrostatin‐1 (Nec‐1), a RIPK1 inhibitor, drastically increased the survival of murine neuronal cells *in vitro* and promoted memory and learning retention as well as cognitive performance in AD models.^8^, ^9^ Nec‐1 treatment was further demonstrated to significantly diminish the level of Aβ oligomers, plaques and hyperphosphorylated tau.^9^ Moreover, Yang et al. proved that Nec‐1 could disaggregate Aβ fibrils and oligomers and block Aβ aggregate‐induced brain cell death.^10^ In SH‐SY5Y cells, RIPK1expression was considerably upregulated after glutamate treatment, suggesting that the protein may specifically regulate programmed necrosis in glutamate‐mediated excitatory toxicity.^11^ Taken together, RIPK1 showed to play an important role in AD. However, evidence from these studies is insufficient to elucidate the role of RIPK1 in mediating Aβ‐induced cell death and APP processing in human AD models. Hence, potential RIPK1‐mediated cell death mechanisms were investigated in SH‐SY5Y cells treated with Aβ, as well as its role on Aβ‐induced cell death and APP protein stability. The results of this study may prove beneficial to enhance targeted therapies effective for AD in the future.

Emerging documentations showed that human neuroblastoma SH‐SY5Y cells have been used as a cell model of neurodegenerative diseases, including AD. SH‐SY5Y cells in both undifferentiated and differentiated forms express a number of neuronal markers such as tyrosine hydroxylase, muscarinic and nicotinic acetylcholine receptors, that are frequently used in *in vitro* neurological experiments.^12^ In order to establish a human AD cell model, the undifferentiated SH‐SY5Y cells are often challenged with Aβ or stably overexpressing APP to mimic the AD phenotypes *in vitro*.^13^, ^14^, ^15^, ^16^

## 2 MATERIALS AND METHODS

### 2.1 Cell culture

Human embryonic kidney 293T (HEK‐293T) cells and human neuroblastoma SH‐SY5Y cells were obtained from American Type Culture Collection (ATCC; Manassas, VA, USA). The cells were cultured in Dulbecco's Modified Eagle Medium (DMEM) (Corning Inc.) supplemented with 10% foetal bovine serum (FBS) (Biosera), penicillin 100 IU/mL and streptomycin 100 µg/ml (Sigma‐Aldrich). The cells were cultured in an incubator with 5% carbon dioxide at 37°C and passaged upon reaching 80% confluence.

### 2.2 Lentivirus production and transduction

APP expression lentiviral and control constructs were obtained from GeneCopoeia (Rockville, MD, USA). RIPK‐1 short hairpin RNA (shRNA) lentiviral constructs (pLKO.1‐puro vector, RIPK‐1‐si‐1 and RIPK‐1‐si‐2) were obtained from Sigma‐Aldrich. The RIPK1 expression lentiviral construct was obtained from ABM. Briefly, high‐titre lentiviruses were produced by co‐transfection with packaging plasmids psPAX2 (Addgene plasmid #12260) and envelope plasmids pMD2.G (Addgene plasmid #12259) into HEK‐293T using CalPhos Transfection Kits (Clontech). Supernatants containing lentiviruses were supplemented with polybrene (Sigma) and used for the transduction of SH‐SY5Y cells to generate stable cell lines by brief antibiotic selection with either 1 µg/ml of puromycin or 500 µg/ml of G418. The details of the expression plasmids and target sequence of shRNA plasmids are shown in Tables S1 and S2.

### 2.3 Aβ preparation

Aβ 1–40, Aβ 1–42 and their respective reverse control peptides (Aβ 40–1 and Aβ 42–1) were purchased from Elabscience (Elabscience). The Aβ was reconstituted with acetonitrile‐water (1:5) to 5 mg/ml. To form Aβ oligomers, the peptides were incubated for 72 h at 37°C and incubated for 2 weeks at 4°C to facilitate higher‐order aggregation. The aggregated Aβ peptides were aliquoted and stored at −80°C for future experiments. The oligomerisation status of the peptide was accessed by immunoblotting as described in Stine et al. before experiments.^17^

### 2.4 CellTiter‐Glo luminescent cell viability assay

Cells were seeded in 384‐well plate at a density of 6 × 10^3^ cells per well and treated with Aβ and/or inhibitors (Z‐vad, 3‐Methyladenine (3‐MA), Nec‐1, necrosulfonamide (NSA), GSK‐872) as described in Table S3 and Table S4 for 72 h. Briefly, CellTiter‐Glo^®^ substrate and buffer (Promega Corporation) were mixed to form a homogenous solution. Prior adding to the cells, the mixture was equilibrated to room temperature and the required amount was diluted at 1:1 ratio with phosphate‐buffered saline (PBS). The mixture was then added at 1:1 ratio to each well and mixed on an orbital shaker. The plate was left in room temperature for 10 min to stabilise the luminescent signal, which was subsequently recorded using the SoftMax Pro 6.3 application on the SpectraMax microplate reader.

### 2.5 Caspase assay

Cells were seeded in 384‐well plate at a density of 6 × 10^3^ cells per well and treated with 80 µM of Aβ. Caspase catalytic activity was determined at 72 h post‐treatment using Caspase‐Glo 3/7, Caspase‐Glo 8 and Caspase‐Glo 9 Assay kits (Promega) according to the manufacturer's instructions. The signal was recorded using the SoftMax Pro 6.3 application on the SpectraMax microplate reader.

### 2.6 Protein stability assay

Cells were seeded in 6‐well plate at a density of 6 × 10^5^ cells per well. Following overnight incubation, the cells were treated with cycloheximide (CHX) (Cayman Chemicals) at a concentration of 10 µg/ml. Protein lysate was harvested and quantified at 0, 30, 60 and 90 min after treatment. The expression of RIPK1 and APP was determined by immunoblotting.

### 2.7 Immunoblotting assay

Protein lysates were extracted using ice‐cold lysis buffer (1% NP‐40, 1mM DTT, supplemented with protease and phosphatase inhibitors in PBS) and subjected to immunoblotting. Primary antibodies against APP and β‐actin were obtained from Biolegend, and Santa Cruz Biotechnology Inc, respectively. RIP, phospho‐RIP, MLKL, phospho‐MLKL, RIP3 and phospho‐RIP3 were purchased from Cell Signalling Technology Inc. The dilution ratio used for each antibody was shown in Table S5. All images were captured using the ChemiDocTM XRS+molecular imager (Bio‐Rad Laboratories).

### 2.8 Quantitative real‐time PCR analysis

Total RNA extraction and conversion of RNA to cDNA was performed using QIAGEN RNeasy Mini Kit (Qiagen) and High Capacity RNA‐to‐cDNA Kit (Applied Biosystems) respectively, according to instruction manual. PCR amplification was carried out with the CFX96 Touch Real Time System (Bio‐Rad Laboratories). The conditions set for RT‐qPCR were as follows: 2 min at 94°C followed by a total 50 cycles of 20 s at 94°C, 30 s at 60°C and 30 s at 72°C. GAPDH served as a housekeeping gene for normalisation. All the experiments were performed in duplicate. The specific forward and reverse primer sequences used are shown in Table S6.

### 2.9 Statistical analysis

All data were expressed as mean ± standard deviation from at least three independent experiments. The analysis was performed in Student's *t* test with Microsoft Excel, to compare the differences between two groups and one‐way ANOVA, followed by Tukey's multiple comparison test with SPSS software version 28.0 (IBM SPSS), to compare the differences in more than two groups. Data with *p *< 0.01 were considered statistically significant.

## 3 RESULTS

### 3.1 Aβ 1–42 had a higher tendency to form oligomers

**FIGURE 1:**
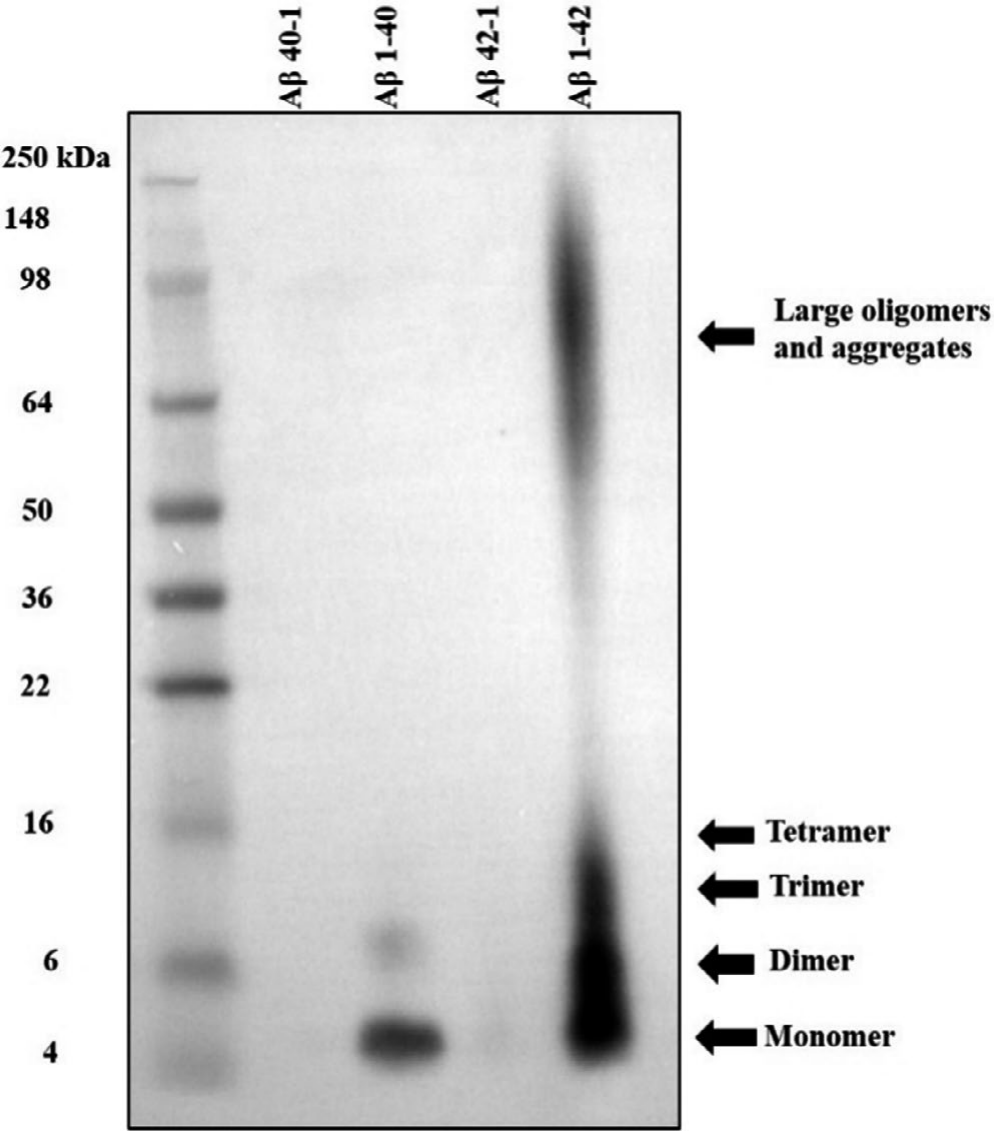
Aβ 1–42 tends to form large aggregate peptides compared to Aβ 1–40. Aβ peptides were reconstituted with acetonitrile‐water (1:5) in 5 mg/ml. The reconstituted peptides were incubated at 37°C for 72 h and 4°C for two weeks. Immunoblotting was performed to validate the aggregation status of Aβ. Reverse control peptides were included

To investigate the tendency of Aβ 40 and Aβ 42 to form oligomer or aggregates, both Aβs were reconstituted and incubated under the same conditions. As shown in Figure 1, Aβ 1–42 tended to produce large oligomers and aggregates, whilst Aβ 1–40 was mainly in its monomer or dimer forms (4–8 kDa). On the contrary, their corresponding reverse control peptides (Aβ 40–1 and Aβ 42–1) did not form aggregates.

### 3.2 Aβ dose‐dependently inhibited cell viability in SH‐SY5Y cells

**FIGURE 2:**
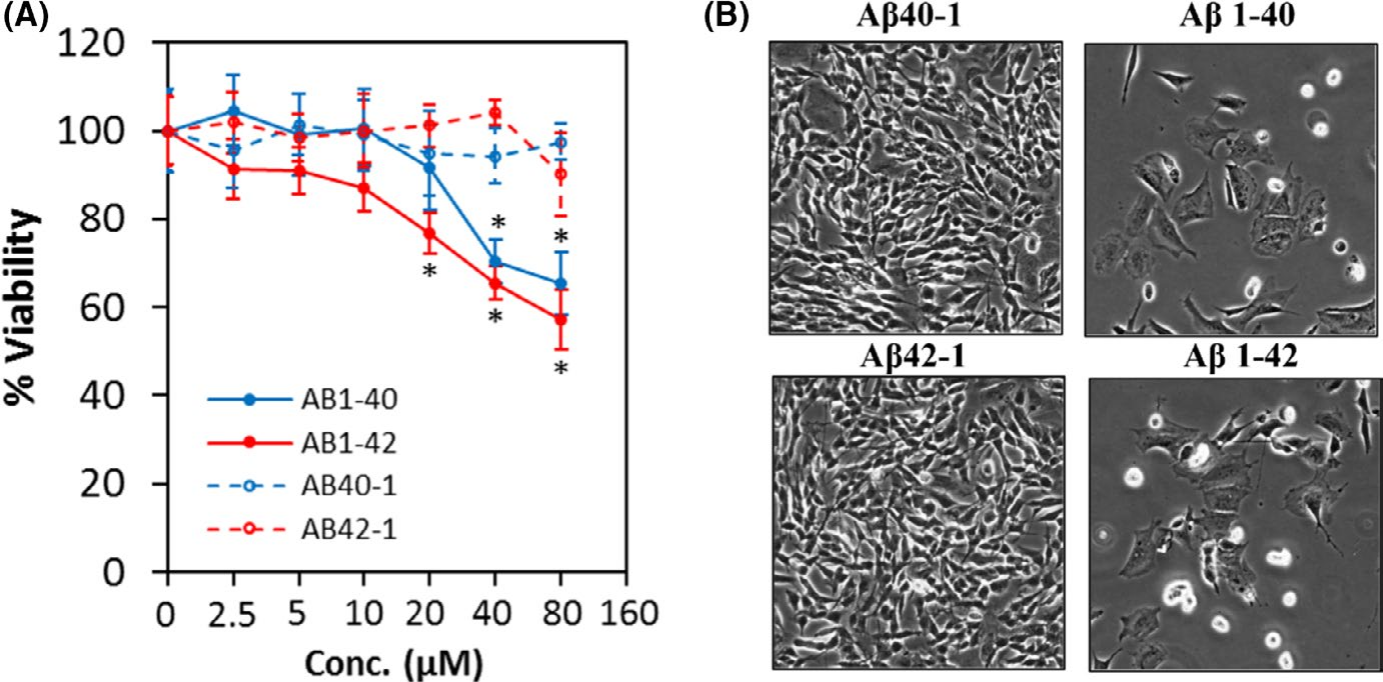
Aβ reduces cell viability and causes morphological changes. (A) Cell viability of Aβ 40–1, Aβ 1–40, Aβ 42–1 and Aβ 1–42 treated cells at different concentrations at 72 h. All data represent the mean ± standard deviation. Asterisks (*) indicate statistical significance (*p* < 0.01), compared with control group. (B) Morphological changes were observed in cells treated with respective Aβ at 80 µM at 72 h. Images were taken at 100× magnification

To investigate the neurotoxic effect of Aβ in SH‐SY5Y cells, concentrations from 0 µM to 80 µM were used. The cell viability assay showed that both Aβ 1–40 and Aβ 1–42 decreased cell viability in a dose‐dependent manner up to 80 µM (Figure 2A), whereas their corresponding reverse control peptides (Aβ 40–1 and Aβ 42–1) did not exert the same effect. Treatment with Aβ 1–42 attenuated cell viability at concentrations as low as 20 µM compared to Aβ 1–40 (40 µM), suggesting that Aβ 1–42 is more neurotoxic compared to Aβ 1–40.

Under microscope observation, crystal like‐structures were observed on cells treated with 80 µM of Aβ 1–40 or Aβ 1–42. Due to the concern of whether the cells could uptake Aβ in the saturated environment, a dose that caused approximately 50% of cell viability reduction (80 µM) was chosen. Hence, the concentration of 80 µM was used for further experiments. Morphologically, cells treated with Aβ 1–40 or Aβ 1–42 at 80 µM transformed from its bipolar‐elongated shape into an irregular‐polygonal shape, whereas no change was observed in cells treated with reverse control peptides (Figure 2B).

### 3.3 Aβ induces caspase‐independent neuronal cell death

**FIGURE 3:**
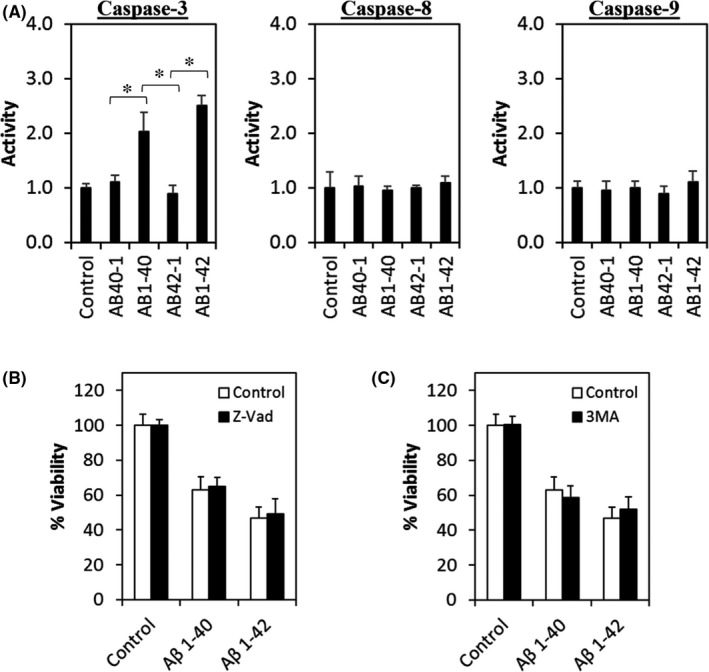
Aβ‐induced neuronal cell death is independent of caspase activity and autophagy. (A) Caspase‐3, caspase‐8 and caspase‐9 activities were measured after treating the cells with Aβ at 72 h. (B) Cell viability of Aβ co‐treated with Z‐Vad (10 µM) or Aβ alone and control group at 72 h. (C) Cell viability of Aβ co‐treated with 3‐MA (100 µM) or Aβ alone and control group at 72 h. All data represent the mean ± standard deviation. Asterisks (*) indicate statistical significance (*p* < 0.01), compared with control group

In this study, cells treated with Aβ were subjected to caspase activity assay. Figure 3A shows that the activity of apoptosis initiator caspase‐8 and caspase‐9 was no different between the Aβ‐treated groups, reverse control peptide groups and control group (cell with culture medium only). Surprisingly, the activity of executioner caspase‐3 was significantly increased in both Aβ 1–40 and Aβ 1–42, compared with their corresponding reverse control peptide group and control group.

To further investigate whether the neuronal cell death was dependent on caspase activity, cells were co‐treated with Aβ and 10 µM caspase inhibitor Z‐Vad. Figure 3B shows that Z‐Vad did not exert any neuroprotective effect by reversing the cell viability treated with Aβ, compared to cells treated with Aβ alone and control group. This finding suggests that Aβ induces caspase‐independent neuronal cell death.

Since autophagy has been reportedly involved in AD, the role of autophagy in Aβ‐induced neuronal cell death was assessed. As shown in Figure 3C, cells co‐treated with Aβ and 10 mM of 3‐MA, an autophagy inhibitor, did not improve the cell viability as compared to cells treated with Aβ alone, implying that autophagy may not be involved in mediating Aβ‐induced neuronal cell death.

### 3.4 Aβ and APP overexpression activate necroptosis

**FIGURE 4:**
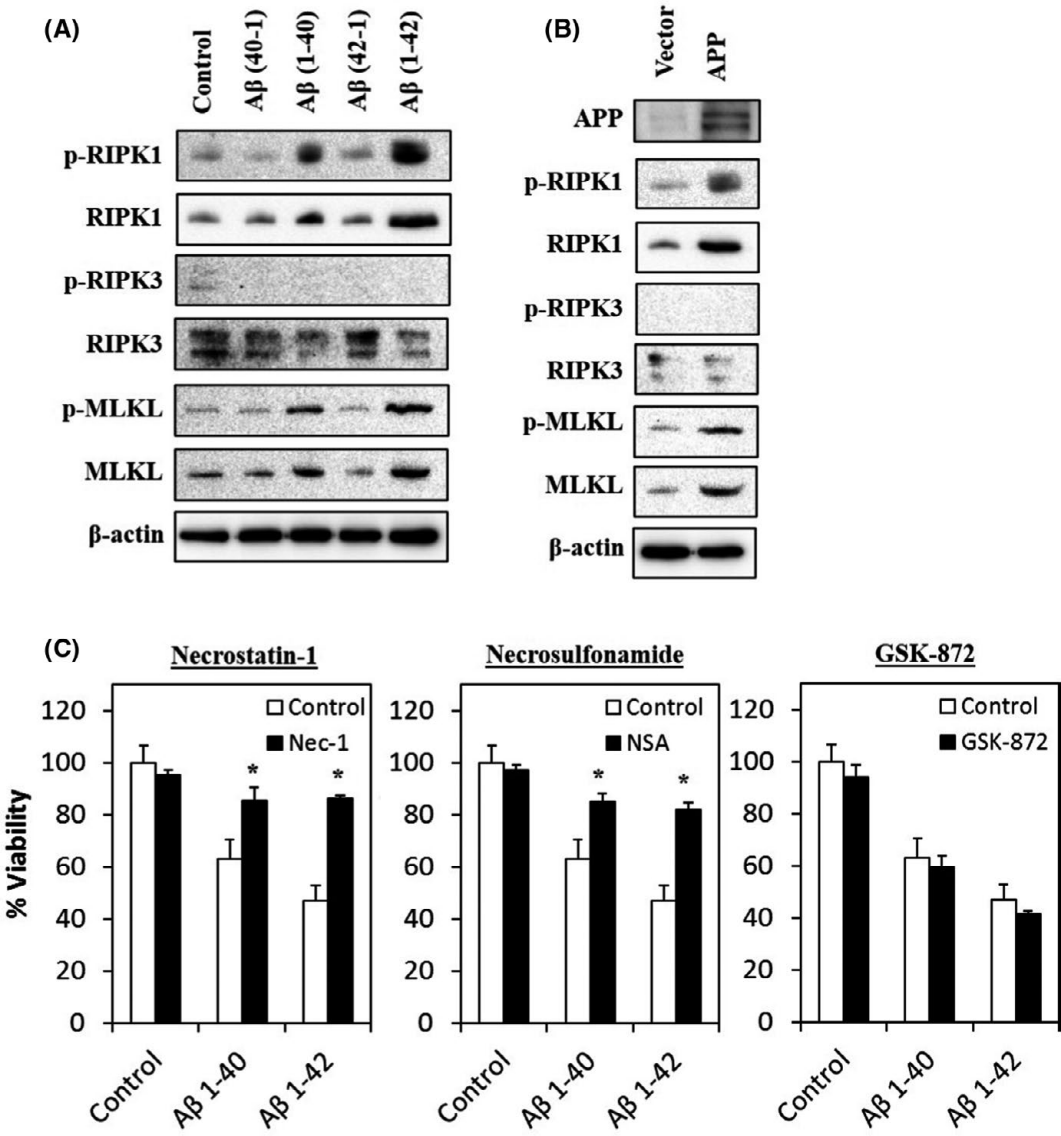
Necroptosis is activated in Aβ‐treated and APP‐overexpressed cells. (A) Necroptosis‐related protein expression was detected by immunoblotting after treatment with Aβ for 72 h. (B) APP and necroptotic‐related proteins expression were detected by immunoblotting in cells overexpressing APP. (C) Cell viability of Aβ co‐treated with necrostatin‐1 (10 µM), necrosulfonamide (1 µM) or GSK‐872 (5 µM); or Aβ alone and control group at 72 h. All data represent the means ± standard deviation. Asterisks (*) indicate statistical significance (*p* < 0.01), compared with control group

The role of Aβ and APP in the activation of necroptosis was assessed by evaluating their protein expression in Aβ‐treated cells through immunoblotting. As shown in Figure 4A, the protein expression of RIPK1, p‐RIPK1, MLKL and p‐MLKL was upregulated in Aβ‐treated cells, whilst the expression of RIPK‐3 was downregulated, compared with cells treated with reverse control peptide or culture medium only (control). However, the expression of p‐RIPK3 was barely detectable across the control and treated groups.

The expression of necroptosis‐related proteins was determined in APP‐overexpressed cells. As expected, the protein expression of RIPK1, p‐RIPK1, RIPK3, p‐RIPK3, MLKL and p‐MLKL showed a similar expression trend in cells treated with Aβ (Figure 4B), suggesting that Aβ and APP are capable of activating necroptotic‐cell death in AD.

To further validate the role of Aβ in the activation of necroptosis, a rescue experiment was performed by co‐treating the Aβ‐treated cells with necroptotic‐inhibitors. Figure 4C shows that Nec‐1, a RIPK1 inhibitor and necrosulfonamide, a MLKL inhibitor, markedly rescued the Aβ‐treated cells from neurotoxicity compared to cells treated with Aβ alone and control group. However, RIPK3 inhibitor (GSK‐872) did not show any neuroprotection in Aβ‐induced neuronal cell death, instead causing further reduction of cell viability. However, the difference was not significant.

### 3.5 RIPK‐1 is required for Aβ‐induced neuronal cell death

**FIGURE 5:**
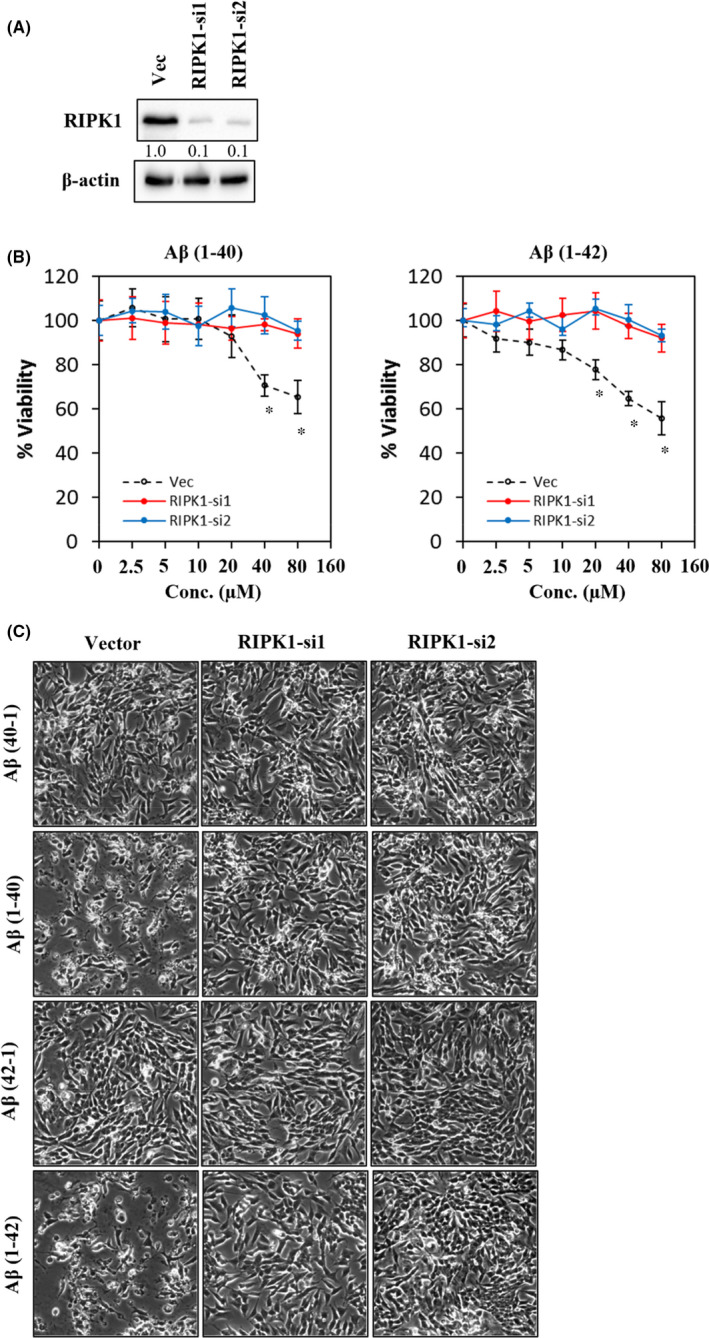
Inhibition of RIPK1 expression protects cells from Aβ‐induced cell death. (A) Efficient inhibition of RIPK1 expression was attained by two independent shRNA constructs. Vector (Vec) was included as control for validation of knockdown efficiency. (B) Cell viability of Aβ 1–40 and Aβ 1–42 treated cells at different concentrations at 72 h. All data represent the mean ± standard deviation. Asterisks (*) indicate statistical significance (*p* < 0.01), compared with control group. (C) Morphological images were captured in cells treated with respective Aβ at 80 µM at 72 h. Images were taken at 100x magnification

In the present study, the role of RIPK1 in mediating Aβ‐induced neuronal cell death was demonstrated in a human cell model. Briefly, inhibition of RIPK1 protein expression was achieved by two independent shRNA constructs. Two RIPK1 shRNA constructs were used to prevent any off‐target effects (Figure 5A). The role of RIPK1 in mediating Aβ‐induced neuronal cell death was investigated by treating the cells with Aβ at concentrations of up to 80 µM. Depletion of RIPK1 expression was able to inhibit neuronal cell death induced by both Aβ 1–40 and Aβ 1–42 (Figure 5B), suggesting that RIPK1 is required for Aβ‐induced neuronal cell death. Furthermore, RIPK1‐deficient cells treated with 80 µM Aβ did not show any morphological changes, compared to their respective reverse control peptide (Figure 5C).

### 3.6 RIPK1 mediation of Aβ‐induced neuronal cell death is independent of caspase activity

**FIGURE 6:**
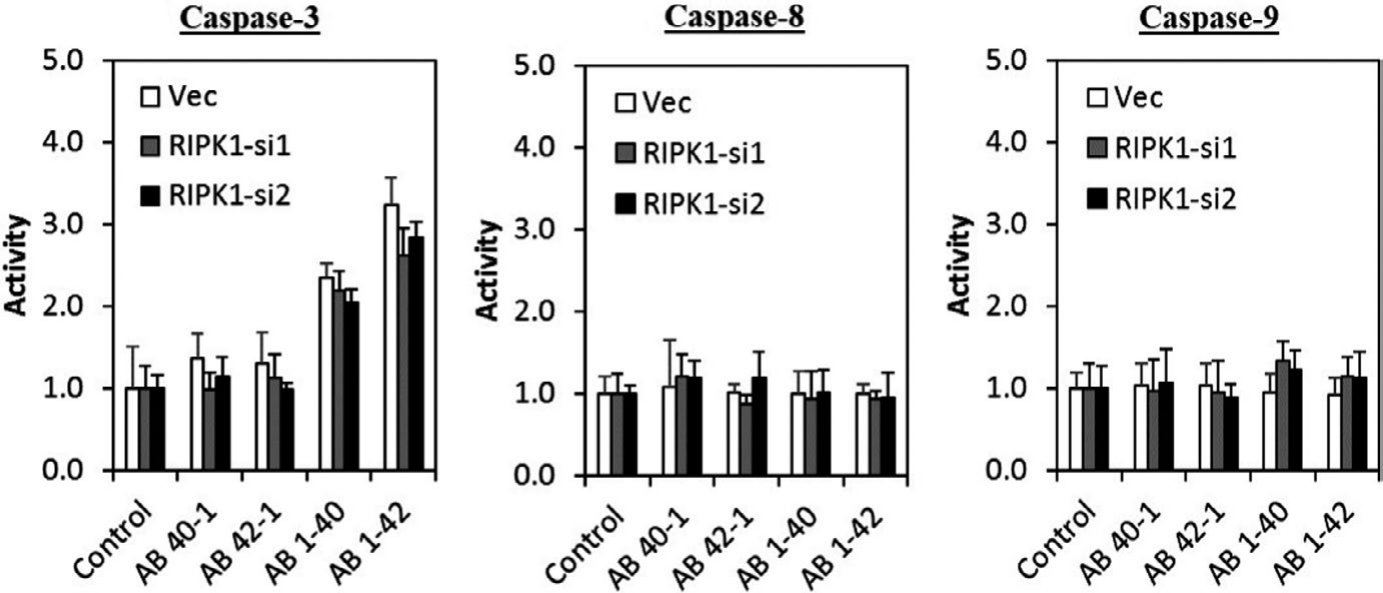
RIPK1 mediates Aβ‐induced neuronal cell death is independent of caspase activity. Caspase‐3, caspase‐8 and caspase‐9 activities were measured after treating the cells with respective Aβ at 80 µM at 72 h

To confirm whether the RIPK1 is involved in mediating Aβ‐induced neuronal cell death *via* apoptosis, RIPK1‐deficient cells were treated with Aβ at 80 µM for 72 h and subjected to a caspase activity assay. Figure 6 showed that the activity of all three caspases was not significantly different between the RIPK1‐deficient cells and its control (Vec), suggesting that RIPK1 mediates Aβ‐induced neuronal cell death through a caspase‐independent mechanism.

### 3.7 Endogenous APP protein stability is dependent on RIPK1

**FIGURE 7:**
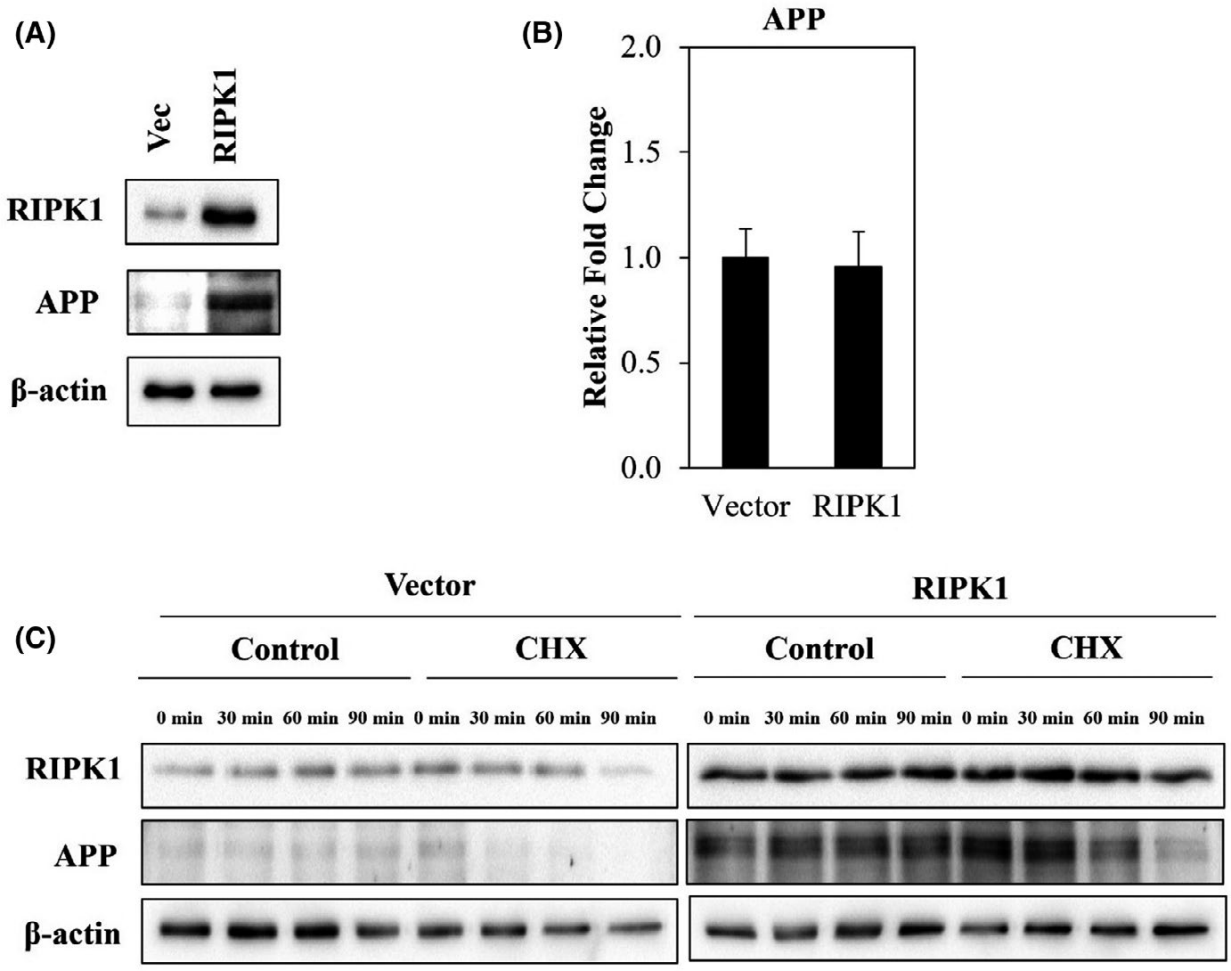
RIPK1 stabilises endogenous APP protein independent of APP mRNA transcription. (A) RIPK1 was overexpressed in cells. Vector (Vec) was included as control for validation of overexpression efficiency. (B) mRNA expression of APP in cells overexpressing RIPK1. All data were normalised against GAPDH and represent the mean ± standard deviation. (C) RIPK1 increased the lifespan of endogenous APP. Cells were treated with 10 µg/mL CHX at 0, 30, 60 and 90 min

In this study, cells overexpressing RIPK1 were generated. As shown in Figure 7A, protein expression of endogenous APP was increased in cells overexpressing RIPK1. Surprisingly, qPCR results showed that the expression of endogenous APP did not show any differences compared with vector control (Figure 7B), suggesting that induction of endogenous APP protein expression does not depend on its mRNA transcriptional level. Additionally, the cycloheximide (CHX) chase assay showed that RIPK1 noticeably increased the lifespan of endogenous APP (Figure 7C), suggesting that RIPK1 stabilises endogenous APP rather than transcriptional activation.

## 4 DISCUSSION

In the present study, we demonstrated that RIPK1 expression was significantly upregulated in cells treated with Aβ or cells overexpressing APP, which may lead to the development of necroptosis‐mediated neuronal cell death in AD. Furthermore, inhibition of RIPK1 appears to be one of the underlying contributors to cell survival against Aβ‐induced neurotoxicity. We further demonstrated that upregulation of RIPK1 enhanced the stability of endogenous APP. Our data suggest that modulation of RIPK1 expression would have a significant impact on future therapeutic strategies for AD treatment.

Multiple lines of evidence have shown that RIPK1 is involved in modulating numerous signalling pathways in neuronal cells such as apoptosis, autophagy and necroptosis.^18^, ^19^, ^20^, ^21^, ^22^, ^23^, ^24^ Apoptosis has been studied intensively in AD, but the outcomes remained contradictory.^25^, ^26^, ^27^, ^28^, ^29^, ^30^, ^31^, ^32^, ^33^ In this study, increased caspase‐3 activity was observed in cells treated with Aβ, whilst the activities of caspase‐8 and caspase‐9 were unchanged.

To further investigate the role of increased caspase‐3 activity, we co‐treated the cells with Aβ and caspase inhibitor (Z‐Vad) and the results showed that co‐treatment with Z‐vad did not significantly improve cell viability. Thus, these findings indicate that the neurotoxic effect of Aβ is independent of caspase activity. Aside from apoptosis, activation of caspase‐3 has been shown to process and activate pro‐interleukin‐16 and stimulate T‐lymphocytes.^34^, ^35^ Perhaps, increased caspase‐3 activity in the current study was involved in activating the inflammatory process rather than apoptosis. On the contrary, autophagy is a bulk degradation pathway for large and aggregated proteins such as Aβ, and has also been implicated in AD.^36^, ^37^, ^38^, ^39^ However, current findings show that co‐treatment of Aβ‐treated cells with autophagy inhibitor (3‐MA) did not show any rescue effects on cell viability.

Necroptosis is a programmed form of necrosis in mediating cell death and was first documented as a consequence of inflammation.^40^ Receptor‐interacting protein kinase 3 (RIPK‐3) and its substrate, the pseudokinase mixed lineage kinase domain‐like protein (MLKL), are core components of the necroptotic signalling pathway.^41^ RIPK1 is known to be involved in RIPK‐3‐MLKL‐dependent necroptosis.^42^, ^43^ Recently, activated necrosomes consisting of p‐RIPK1, p‐RIPK3 and p‐MLKL were found in granulovacuolar degeneration lesions in the degenerating neurons of preclinical AD and AD patients.^23^ However, there is no concrete evidence to support that necroptosis‐mediated neuronal cell death is induced by Aβ.

Hence, the expression level of necroptotic‐related proteins was investigated in current study, in both Aβ‐treated and APP‐overexpressed AD cell models. Our findings revealed that the expression levels of RIPK1, p‐RIPK1, MLKL and p‐MLKL were increased after exposure to Aβ. A similar observation was attained from ectopic expression of APP. Further experiments showed that inhibition of RIPK1 and MLKL by pharmacological means markedly increased the viability of Aβ‐treated cells. A similar study reported that both Nec‐1 and NSA protected human embryonic stem cell‐derived motor neurons (hES‐MNs) from human sporadic ALS astrocyte toxicity.^44^ Furthermore, inhibition of MLKL by NSA was able to reverse the interleukin‐1β‐induced nucleus pulposus cell death, suggesting that NSA could protect intervertebral disc degeneration *via* necroptosis and apoptosis inhibition.^45^ A study conducted by Qiu et al. showed that inhibition of RIPK1 expression could activate apoptosis by cleaving the caspase‐3; however, these findings are contradicted to the current study whereby the expression of RIPK1 and activity of caspase‐3 were upregulated after the cells treated with Aβ.^46^ More efforts are needed to investigate the role of caspase‐3 and RIPK1 in mediating the necroptosis.

Although RIPK1 is known to be a death receptor that activates RIPK3 and MLKL to mediate necroptosis,^47^ contradictory findings indicated that RIPK1 could intrinsically suppress spontaneous RIPK3 activation in the cytosol by controlling RIPK3 oligomerisation.^48^ Furthermore, p‐MLKL has been reported to activate necroptosis in the absence of death stimuli, and this observation was most profound in MLKL and RIPK3 double knockdown cells, suggesting that RIPK3 is a suppressor of MLKL activation.^49^ Zhang et al.^50^ showed that the lipopolysaccharide (LPS)‐induced acute kidney injury increased tubular epithelial cell apoptosis and RIPK3 expression in mice. Conversely, inhibition of RIPK3 was shown to reduce the apoptosis in tubular epithelial cells and improve renal function in mice with LPS‐induced acute kidney injury.^50^

In cancer biology, knockdown of RIPK3 was reported to trigger p53 signalling and mitotic defects in murine recurrent tumour cells.^51^ Furthermore, silencing of RIPK3 in recurrent tumour cells caused depletion of YAP and TAZ (transcriptional coactivator with PDZ‐binding motif), which are essential in mediating the expression levels of proliferation‐promoting and cancer‐causing genes, indicating that RIPK3 might be responsible to the proliferation and recurrence of tumour cells^51^, ^52^ In the present study, we showed that the expression of RIPK‐3 was decreased in both of the Aβ‐treated and APP‐overexpressed AD cell models, and inhibition of its activity by pharmacological means in Aβ‐treated cells did not ‘rescue’ cell viability. Thus, the mechanism underlying the Aβ‐induced neuronal cell death may be far more complex than previously thought. Further efforts are indeed necessary to elucidate the underlying molecular mechanism of RIPK3 in activating necroptosis induced by Aβ in the presence of RIPK1.

Emerging evidence indicates that Nec‐1 displays a neuroprotective effect in reversing the clinical hallmarks and symptoms in a murine AD model.^7^, ^8^, ^9^, ^10^ However, these beneficial outcomes have yet to be elucidated on a human AD model. In this study, RIPK1‐deficient cells were resistant to the cytotoxicity induced by Aβ, and its effect was independent of caspase activity. In accordance with our data, Re et al. demonstrated that depletion of RIPK1 protected human embryonic stem cell‐derived motor neurons against sporadic ALS astrocyte toxicity.^44^ Furthermore, Yang et al. showed that co‐treatment of Nec‐1 in Aβ‐treated HT22 cells did not affect the expression of cleaved caspase‐3.^9^ However, Nec‐1 has been shown to reduce the expression of cleaved caspase‐3 in non‐neuronal cells such as mouse microglial cells (BV2) and primary astrocytes treated with Aβ.^9^ These contradicting outcomes suggest that RIPK1‐mediated necroptosis may be cell‐type specific, with a tendency to affect neuronal cells.

Aβ monomers have the ability to form oligomers and fibrils during the progression of AD. A recent study conducted by Yang et al. showed that Nec‐1 was found to disaggregate Aβ fibrils and oligomers and inhibit brain cell death.^10^ In the mentioned study, thioflavin‐T fluorescence assay was performed to quantify the aggregation of Aβ.^10^ Results showed that Nec‐1 significantly reduced Aβ fibrils.^10^ Furthermore, cytotoxicitiy assay of pre‐formed Aβ 1–42 aggregates in cells revealed that Nec‐1 demonstrated neuroprotective effect against Aβ aggregates, proposing that such anti‐neuronal cell death effects were contributed by Nec‐1 in dissociating Aβ aggregates.^10^ An *in vivo* study also showed that Nec‐1 was able to diminish Aβ plaques in the brains of aged APP/PS1 mice.^10^ However, data on the roles of RIPK1 in the Aβ precursor protein are highly limited. In accordance with a previous study, our findings indicate that ectopic expression of RIPK1 increases the expression and stability of endogenous APP protein independent of mRNA expression. Hence, we postulate that RIPK1 is involved in the regulation of endogenous APP stability and perhaps acts to accelerate AD progression.

## 5 CONCLUSION

In summary, we demonstrated that Aβ‐induced neuronal cell death involves an RIPK1/MLKL‐dependent necroptosis mechanism in an *in vitro* human AD model. Furthermore, the expression of RIPK1 was significantly increased in both Aβ‐treated and APP‐overexpressed AD cell models. Additionally, inhibition of RIPK1 expression rescued the cells from Aβ‐induced neuronal cell death, whilst ectopic expression of RIPK1 enhanced the stability of endogenous APP. Contrary to the canonical necroptosis pathway, Aβ‐induced RIPK1‐MLKL‐dependent necroptosis is independent of RIPK3 activity. Hence, these findings suggest that RIPK1 could be a potential target in reversing the pathogenesis of AD.

## 6 FUTURE PROSPECTS

Although current findings showed that RIPK1 mediated Aβ‐induced neuronal cell death in RIPK1‐MLKL‐depedent necroptosis and independent of RIPK3 activity, however, as forementioned the neuropathological hallmarks of AD include the deposition of extracellular Aβ and intracellular neurofibrillary tau proteins. Perhaps in the future study, the role of RIPK1 in tau protein‐induced neuronal cell death needs to be investigated. In addition to neuronal cells, participating cells in the brain include astrocytes, oligodendrocytes and microglial are important in maintaining brain homeostasis and function. Therefore, developing a 3D‐culture or coculture system is needed to investigate the role of RIPK1 in the pathogenesis of AD. Lastly, further downstream assays are needed to explain the increased caspase‐3 observed in current study and elucidate the interaction of RIPK1, RIPK3, MLKL and caspase‐3 in mediating necroptosis in AD.

## CONFLICT OF INTEREST

The author(s) declare no competing interests.

## AUTHOR CONTRIBUTIONS

**Hong Hao Chan:** Data curation (equal); Formal analysis (equal); Investigation (equal); Methodology (equal); Writing – original draft (equal). **Chee‐Onn Leong:** Conceptualization (equal); Data curation (equal); Formal analysis (equal); Funding acquisition (equal); Investigation (equal); Methodology (equal); Project administration (equal); Supervision (equal); Validation (equal); Visualization (equal); Writing – review & editing (equal). **Chooi Ling Lim:** Funding acquisition (equal); Investigation (equal); Methodology (equal); Project administration (equal); Supervision (equal); Validation (equal); Writing – review & editing (equal). **Rhun Yian Yian Koh:** Conceptualization (equal); Funding acquisition (equal); Investigation (equal); Methodology (equal); Project administration (equal); Supervision (equal); Validation (equal); Writing – original draft (equal); Writing – review & editing (equal).

## Supporting information

Table S1‐S6

## Data Availability

The data that support the findings of this study are available in the supplementary material of this article.

## References

[1] Swerdlow RH . Pathogenesis of Alzheimer's disease. Clin Interv Aging. 2007;2(3):347‐359. 18044185 PMC2685260

[2] Chow VW , Mattson MP , Wong PC , Gleichmann M . An overview of APP processing enzymes and products. NeuroMol Med. 2010;12(1):1‐12. 10.1007/s12017-009-8104-z PMC2889200 20232515

[3] Caccamo A , Oddo S , Sugarman MC , Akbari Y , LaFerla FM . Age‐ and region‐dependent alterations in Abeta‐degrading enzymes: implications for Abeta‐induced disorders. Neurobiol Aging. 2005;26(5):645‐654. 15708439 10.1016/j.neurobiolaging.2004.06.013

[4] Apelt J , Ach K , Schliebs R . Aging‐related down‐regulation of neprilysin, a putative beta‐amyloid‐degrading enzyme, in transgenic Tg2576 Alzheimer‐like mouse brain is accompanied by an astroglial upregulation in the vicinity of beta‐amyloid plaques. Neurosci Lett. 2003;339(3):183‐186. 12633883 10.1016/s0304-3940(03)00030-2

[5] Li J , McQuade T , Siemer AB , et al. The RIP1/RIP3 necrosome forms a functional amyloid signaling complex required for programmed necrosis. Cell. 2012;150(2):339‐350. 22817896 10.1016/j.cell.2012.06.019 PMC3664196

[6] Wegner KW , Saleh D , Degterev A . Complex pathologic roles of RIPK1 and RIPK3: moving beyond necroptosis. Trends Pharmacol Sci. 2017;38(3):202‐225. 28126382 10.1016/j.tips.2016.12.005 PMC5325808

[7] Ofengeim D , Mazzitelli S , Ito Y , et al. RIPK1 mediates a disease‐associated microglial response in Alzheimer's disease. Proc Natl Acad Sci USA. 2017;114(41):E8788‐E8797. 28904096 10.1073/pnas.1714175114 PMC5642727

[8] Qinli Z , Meiqing L , Xia J , et al. Necrostatin‐1 inhibits the degeneration of neural cells induced by aluminum exposure. Restorative Neurology and Neuroscience. 2013;31(5):543‐555. 23735313 10.3233/RNN-120304

[9] Yang SH , Lee DK , Shin J , et al. Nec‐1 alleviates cognitive impairment with reduction of Abeta and tau abnormalities in APP/PS1 mice. EMBO Mol Med. 2017;9(1):61‐77. 27861127 10.15252/emmm.201606566 PMC5210088

[10] Yang SH , Shin J , Shin NN , et al. A small molecule Nec‐1 directly induces amyloid clearance in the brains of aged APP/PS1 mice. Sci Rep. 2019;9(1):4183. 30862818 10.1038/s41598-019-40205-5 PMC6414664

[11] Sun X , Shi X , Lu L , Jiang Y , Liu B . Stimulus‐dependent neuronal cell responses in SH‐SY5Y neuroblastoma cells. Mol Med Rep. 2016;13(3):2215‐2220. 26781445 10.3892/mmr.2016.4759

[12] Kovalevich J , Langford D . Considerations for the use of SH‐SY5Y neuroblastoma cells in neurobiology. Methods Mol Biol. 2013;1078:9‐21. 23975817 10.1007/978-1-62703-640-5_2 PMC5127451

[13] Zamani E , Parviz M , Roghani M , Hosseini M , Mohseni‐Moghaddam P , Nikbakhtzadeh M . Netrin‐1 protects the SH‐SY5Y cells against amyloid beta neurotoxicity through NF‐kappaB/Nrf2 dependent mechanism. Mol Biol Rep. 2020;47(12):9271‐9277. 33206363 10.1007/s11033-020-05996-1

[14] Shin JY , Park HJ , Kim HN , et al. Mesenchymal stem cells enhance autophagy and increase beta‐amyloid clearance in Alzheimer disease models. Autophagy. 2014;10(1):32‐44. 24149893 10.4161/auto.26508 PMC4389879

[15] Liu M , Bai X , Yu S , et al. Ginsenoside re inhibits ROS/ASK‐1 dependent mitochondrial apoptosis pathway and activation of Nrf2‐antioxidant response in beta‐amyloid‐challenged SH‐SY5Y cells. Molecules. 2019;24(15):2687. 10.3390/molecules24152687 PMC6696356 31344860

[16] Grimm MOW , Blumel T , Lauer AA , et al. The impact of capsaicinoids on APP processing in Alzheimer's disease in SH‐SY5Y cells. Sci Rep. 2020;10(1):9164. 32514053 10.1038/s41598-020-66009-6 PMC7280252

[17] Stine WB , Jungbauer L , Yu C , LaDu MJ . Preparing synthetic Abeta in different aggregation states. Methods Mol Biol. 2011;670:13‐32. 20967580 10.1007/978-1-60761-744-0_2 PMC3752843

[18] Ikner A , Ashkenazi A . TWEAK induces apoptosis through a death‐signaling complex comprising receptor‐interacting protein 1 (RIP1), Fas‐associated death domain (FADD), and caspase‐8. Int J Biol Chem. 2011;286(24):21546‐21554. 10.1074/jbc.M110.203745 PMC3122213 21525013

[19] Wagner L , Marschall V , Karl S , et al. Smac mimetic sensitizes glioblastoma cells to Temozolomide‐induced apoptosis in a RIP1‐ and NF‐kappaB‐dependent manner. Oncogene. 2013;32:988‐997. 22469979 10.1038/onc.2012.108

[20] Yonekawa T , Gamez G , Kim J , et al. RIP1 negatively regulates basal autophagic flux through TFEB to control sensitivity to apoptosis. EMBO Rep. 2015;16(6):700‐708. 25908842 10.15252/embr.201439496 PMC4467854

[21] Luan Q , Jin L , Jiang CC , et al. RIPK1 regulates survival of human melanoma cells upon endoplasmic reticulum stress through autophagy. Autophagy. 2015;11(7):975‐994. 26018731 10.1080/15548627.2015.1049800 PMC4590596

[22] Caccamo A , Branca C , Piras IS , et al. Necroptosis activation in Alzheimer's disease. Nat Neurosci. 2017;20(9):1236‐1246. 28758999 10.1038/nn.4608

[23] Koper MJ , Van Schoor E , Ospitalieri S , et al. Necrosome complex detected in granulovacuolar degeneration is associated with neuronal loss in Alzheimer's disease. Acta Neuropathol. 2020;139(3):463‐484. 31802237 10.1007/s00401-019-02103-y

[24] Zhang X , Dowling JP , Zhang J . RIPK1 can mediate apoptosis in addition to necroptosis during embryonic development. Cell Death Dis. 2019;10(3):245. 30867408 10.1038/s41419-019-1490-8 PMC6416317

[25] Stadelmann C , Deckwerth TL , Srinivasan A , et al. Activation of caspase‐3 in single neurons and autophagic granules of granulovacuolar degeneration in Alzheimer's disease. Evidence for apoptotic cell death. Am J Clin Pathol. 1999;155(5):1459‐1466. 10.1016/S0002-9440(10)65460-0 PMC1866960 10550301

[26] Rohn TT , Rissman RA , Davis MC , Kim YE , Cotman CW , Head E . Caspase‐9 activation and caspase cleavage of tau in the Alzheimer's disease brain. Neurobiol Dis. 2002;11(2):341‐354. 12505426 10.1006/nbdi.2002.0549

[27] Rohn TT , Head E , Nesse WH , Cotman CW , Cribbs DH . Activation of caspase‐8 in the Alzheimer's disease brain. Neurobiol Dis. 2001;8(6):1006‐1016. 11741396 10.1006/nbdi.2001.0449

[28] Gervais FG , Xu D , Robertson GS , et al. Involvement of caspases in proteolytic cleavage of Alzheimer's amyloid‐beta precursor protein and amyloidogenic A beta peptide formation. Cell. 1999;97(3):395‐406. 10319819 10.1016/s0092-8674(00)80748-5

[29] Loo DT , Copani A , Pike CJ , Whittemore ER , Walencewicz AJ , Cotman CW . Apoptosis is induced by beta‐amyloid in cultured central nervous system neurons. Proc Natl Acad Sci USA. 1993;90(17):7951‐7955. 8367446 10.1073/pnas.90.17.7951 PMC47265

[30] Marin N , Romero B , Bosch‐Morell F , et al. Beta‐amyloid‐induced activation of caspase‐3 in primary cultures of rat neurons. Mech Ageing Dev. 2000;119(1–2):63‐67. 11040402 10.1016/s0047-6374(00)00172-x

[31] Gatta V , Drago D , Fincati K , et al. Microarray analysis on human neuroblastoma cells exposed to aluminum, beta(1–42)‐amyloid or the beta(1–42)‐amyloid aluminum complex. PLoS One. 2011;6(1):e15965. 21298039 10.1371/journal.pone.0015965 PMC3029275

[32] Woodhouse A , Dickson TC , West AK , McLean CA , Vickers JC . No difference in expression of apoptosis‐related proteins and apoptotic morphology in control, pathologically aged and Alzheimer's disease cases. Neurobiol Dis. 2006;22(2):323‐333. 16406795 10.1016/j.nbd.2005.11.010

[33] Tillement L , Lecanu L , Papadopoulos V . Further evidence on mitochondrial targeting of beta‐amyloid and specificity of beta‐amyloid‐induced mitotoxicity in neurons. Neurodegener Dis. 2011;8(5):331‐344. 21311166 10.1159/000323264

[34] Miossec C , Dutilleul V , Fassy F , Diu‐Hercend A . Evidence for CPP32 activation in the absence of apoptosis during T lymphocyte stimulation. Int J Biol Chem. 1997;272(21):13459‐13462. 10.1074/jbc.272.21.13459 9153186

[35] Zhang Y , Center DM , Wu DM , et al. Processing and activation of pro‐interleukin‐16 by caspase‐3. Int J Biol Chem. 1998;273(2):1144‐1149. 10.1074/jbc.273.2.1144 9422780

[36] Lucin KM , O'Brien CE , Bieri G , et al. Microglial beclin 1 regulates retromer trafficking and phagocytosis and is impaired in Alzheimer's disease. Neuron. 2013;79(5):873‐886. 24012002 10.1016/j.neuron.2013.06.046 PMC3779465

[37] Jaeger PA , Pickford F , Sun CH , Lucin KM , Masliah E , Wyss‐Coray T . Regulation of amyloid precursor protein processing by the Beclin 1 complex. PLoS One. 2010;5(6):e11102. 20559548 10.1371/journal.pone.0011102 PMC2886067

[38] Salminen A , Kaarniranta K , Kauppinen A , et al. Impaired autophagy and APP processing in Alzheimer's disease: the potential role of Beclin 1 interactome. Prog Neurogibol. 2013;106–107:33‐54. 10.1016/j.pneurobio.2013.06.002 23827971

[39] Pickford F , Masliah E , Britschgi M , et al. The autophagy‐related protein beclin 1 shows reduced expression in early Alzheimer disease and regulates amyloid beta accumulation in mice. J Clin Investig. 2008;118(6):2190‐2199. 18497889 10.1172/JCI33585 PMC2391284

[40] Cho YS , Challa S , Moquin D , et al. Phosphorylation‐driven assembly of the RIP1‐RIP3 complex regulates programmed necrosis and virus‐induced inflammation. Cell. 2009;137(6):1112‐1123. 19524513 10.1016/j.cell.2009.05.037 PMC2727676

[41] Zhang S , Tang MB , Luo HY , Shi CH , Xu YM . Necroptosis in neurodegenerative diseases: a potential therapeutic target. Cell Death Dis. 2017;8(6):e2905. 28661482 10.1038/cddis.2017.286 PMC5520937

[42] Pasparakis M , Vandenabeele P . Necroptosis and its role in inflammation. Nature. 2015;517(7534):311‐320. 25592536 10.1038/nature14191

[43] Weinlich R , Oberst A , Beere HM , Green DR . Necroptosis in development, inflammation and disease. Nat Rev Mol Cell Biol. 2017;18(2):127‐136. 27999438 10.1038/nrm.2016.149

[44] Re DB , Le Verche V , Yu C , et al. Necroptosis drives motor neuron death in models of both sporadic and familial ALS. Neuron. 2014;81(5):1001‐1008. 24508385 10.1016/j.neuron.2014.01.011 PMC3951532

[45] Zhang QX , Guo D , Wang FC , Ding WY . Necrosulfonamide (NSA) protects intervertebral disc degeneration via necroptosis and apoptosis inhibition. Eur Rev Med Pharmacol Sci. 2020;24(5):2683‐2691. 32196619 10.26355/eurrev_202003_20538

[46] Qiu X , Zhuang M , Lu Z , et al. RIPK1 suppresses apoptosis mediated by TNF and caspase‐3 in intervertebral discs. J Transl Med. 2019;17(1):135. 31029152 10.1186/s12967-019-1886-3 PMC6487042

[47] Wang X , Yousefi S , Simon HU . Necroptosis and neutrophil‐associated disorders. Cell Death Dis. 2018;9(2):111. 29371616 10.1038/s41419-017-0058-8 PMC5833577

[48] Orozco S , Yatim N , Werner MR , et al. RIPK1 both positively and negatively regulates RIPK3 oligomerization and necroptosis. Cell Death Differ. 2014;21(10):1511‐1521. 24902904 10.1038/cdd.2014.76 PMC4158689

[49] Tanzer MC , Tripaydonis A , Webb AI , et al. Necroptosis signalling is tuned by phosphorylation of MLKL residues outside the pseudokinase domain activation loop. Biochem J. 2015;471(2):255‐265. 26283547 10.1042/BJ20150678

[50] Zhang S , Li R , Dong W , et al. RIPK3 mediates renal tubular epithelial cell apoptosis in endotoxininduced acute kidney injury. Mol Med Rep. 2019;20(2):1613‐1620. 31257491 10.3892/mmr.2019.10416 PMC6625383

[51] Lin CC , Mabe NW , Lin YT , et al. RIPK3 upregulation confers robust proliferation and collateral cystine‐dependence on breast cancer recurrence. Cell Death Differ. 2020;27(7):2234‐2247. 31988496 10.1038/s41418-020-0499-y PMC7308288

[52] Zanconato F , Cordenonsi M , Piccolo S . YAP/TAZ at the roots of cancer. Cancer Cell. 2016;29(6):783‐803. 27300434 10.1016/j.ccell.2016.05.005 PMC6186419

