# Supplementary material for: Roles of receptor‐interacting protein kinase 1 in SH‐SY5Y cells with beta amyloid‐induced neurotoxicity
Source: J Cell Mol Med. 2022 Feb 2;26(5):1434–44. doi: 10.1111/jcmm.17095 (PMC8899176; doi:10.1111/jcmm.17095)
Supplement: Supplementary file 1 — Table S1‐S6 [file JCMM-26-1434-s001.docx]

**Table S1: The details of expression plasmids**

| Target gene | Catalogue number | Accession number | Insert size |
| --- | --- | --- | --- |
| APP | EX-Z2553-Lv157 | NM_000484 | 2313 bp |
| RIPK-1 | LV288148 | NM_003804 | 2016 bp |

**Table S2: The target sequence of RIPK1 shRNAs**

| shRNAs | Sequence |
| --- | --- |
| TRCN0000000705 (si-1) | CCGGAGGTCATGTTCTTTCAGCTTACTCGAGTAAGCTGAAAGAACATGACCTTTTTT |
| TRCN0000000707 (si-2) | CCGGCAGGCCAATTCCAAGTCATATCTCGAGATATGACTTGGAATTGGCCTGTTTTT |

**Table S3: Treatment groups in investigating the role of RIPK1 in Aβ-induced neurotoxicity**

| Cell lines | Group | Treatment |
| --- | --- | --- |
| SH-SY5Y cells | 1 | Aβ 40-1 (Control peptide) (0, 2.5, 5, 10, 20, 40 and 80 µM) |
|  | 2 | Aβ 1-40 (0, 2.5, 5, 10, 20, 40 and 80 µM) |
|  | 3 | Aβ 42-1 (Control peptide) (0, 2.5, 5, 10, 20, 40 and 80 µM) |
|  | 4 | Aβ 1-42 (0, 2.5, 5, 10, 20, 40 and 80 µM) |
|  |  |  |
| RIPK1-deficient SH-SY5Y cells | 1 | Aβ 40-1 (Control peptide) (0, 2.5, 5, 10, 20, 40 and 80 µM) |
|  | 2 | Aβ 1-40 (0, 2.5, 5, 10, 20, 40 and 80 µM) |
|  | 3 | Aβ 42-1 (Control peptide) (0, 2.5, 5, 10, 20, 40 and 80 µM) |
|  | 4 | Aβ 1-42 (0, 2.5, 5, 10, 20, 40 and 80 µM) |

**Table S4: Co-treatment of Aβ with caspase, autophagy or necroptosis inhibitors**

| Inhibitor | Group | Treatment |
| --- | --- | --- |
| Caspase inhibitor  (Z-Vad) | 1 | Control (untreated cells) |
|  | 2 | Control + Z-Vad (10 µM) |
|  | 3 | Aβ 1-40 (80 µM) |
|  | 4 | Aβ 1-40 (80 µM) + Z-Vad (10 µM) |
|  | 5 | Aβ 1-42 (80 µM) |
|  | 6 | Aβ 1-42 (80 µM) + Z-Vad (10 µM) |
|  |  |  |
| Autophagy inhibitor  (3-MA) | 1 | Control (untreated cells) |
|  | 2 | Control + 3-MA (100 µM) |
|  | 3 | Aβ 1-40 (80 µM) |
|  | 4 | Aβ 1-40 (80 µM) + 3-MA (100 µM) |
|  | 5 | Aβ 1-42 (80 µM) |
|  | 6 | Aβ 1-42 (80 µM) + 3-MA (100 µM) |
|  |  |  |
| RIPK1 inhibitor  (Nec-1) | 1 | Control (untreated cells) |
|  | 2 | Control + Nec-1 (10 µM) |
|  | 3 | Aβ 1-40 (80 µM) |
|  | 4 | Aβ 1-40 (80 µM) + Nec-1 (10 µM) |
|  | 5 | Aβ 1-42 (80 µM) |
|  | 6 | Aβ 1-42 (80 µM) + Nec-1 (10 µM) |
|  |  |  |
| RIPK3 inhibitor  (GSK-872) | 1 | Control (untreated cells) |
|  | 2 | Control + GSK-872 (5 µM) |
|  | 3 | Aβ 1-40 (80 µM) |
|  | 4 | Aβ 1-40 (80 µM) + GSK-872 (5 µM) |
|  | 5 | Aβ 1-42 (80 µM) |
|  | 6 | Aβ 1-42 (80 µM) + GSK-872 (5 µM) |
| MLKL inhibitor  (NSA) | 1 | Control (untreated cells) |
|  | 2 | Control + NSA (1 µM) |
|  | 3 | Aβ 1-40 (80 µM) |
|  | 4 | Aβ 1-40 (80 µM) + NSA (1 µM) |
|  | 5 | Aβ 1-42 (80 µM) |
|  | 6 | Aβ 1-42 (80 µM) + NSA (1 µM) |

**Table S5: Primary antibodies for immunoblotting assay**

| Primary antibodies | Dilution ratio | Catalogue number | Manufacturers |
| --- | --- | --- | --- |
| APP/Aβ | 1:500 | 803001 | Biolegend, San Diego, California, USA |
| RIP | 1:1000 | 3493 | Cell Signalling Technology Inc., Danvers, MA, USA |
| Phospho-RIP | 1:1000 | 65746 | Cell Signalling Technology Inc., Danvers, MA, USA |
| MLKL | 1:1000 | 14993 | Cell Signalling Technology Inc., Danvers, MA, USA |
| Phospho-MLKL | 1:1000 | 91689 | Cell Signalling Technology Inc., Danvers, MA, USA |
| RIP3 | 1:1000 | 13526 | Cell Signalling Technology Inc., Danvers, MA, USA |
| Phospho-RIP3 | 1:1000 | 93654 | Cell Signalling Technology Inc., Danvers, MA, USA |
| β-actin | 1:1000 | SC-47778 | Santa Cruz Biotechnology Inc, Santa Cruz, CA, USA |

**Table S6: Primer sequences**

| Gene | Gene ID | Forward primer | Reverse primer |
| --- | --- | --- | --- |
| APP | NM_000484.4 | 5’TGAACCCCAGATTGCCATGTTCT3’ | 5’TCACCAACTAAGCAGCGGTAGGG3’ |
| GAPDH | NM_002046.7 | 5’ GTCTCCTCTGACTTCAACAGCG 3’ | 5’ ACCACCCTGTTGCTGTAGCCAA 3’ |
